# Reviewed article: Martins CI, Silva CSO, Souza FG, Faria SMC, Silva LR, Camargos MCS. Sociodemographic profile of elderly people cared for at a medium and high complexity hospital in Belo Horizonte, Minas Gerais, Brazil: a cross-sectional study. Epidemiol Serv Saude. 2025:34;e20240254

**DOI:** 10.1590/S2237-96222025v34e20240254.c

**Published:** 2025-09-08

**Authors:** Isabela Thaís Machado de Jesus

**Table:** 

Rating	Excellent	Good	Average	Below average	Poor
Interest	✓				
Quality		✓			
Originality		✓			
Overall		✓			

**Table:** 

Questionnaire	Yes	No	Not applicable
Does the manuscript contain new and significant information to justify publication?	✓		
Does the Abstract (Summary) clearly and accurately describe the content of the article?	✓		
Is the problem significant and concisely stated?	✓		
Are the methods described comprehensively?	✓		
Are the interpretations and conclusions justified by the results?	✓		
Is adequate reference made to other work in the field?	✓		

## Manuscript Structure

Length of article is: Adequate

Number of tables is: Adequate

Number of figures is: Adequate

Please state any conflict(s) of interest that you have in relation to the review of this paper (state “none” if this is not applicable): None

## Comments

Prezado Autores,

Parabenizo a condução e discussão dos dados em alta complexidade da população idosa.

O artigo apresenta dados locais e dados prevalentes em período de 2015-2019, não temos muitos dados em literatura brasileira sobre hospitalizações, esse parece uma boa caracterização de pessoas idosas em uma capital mineira. Acredito que faltou discutir aspectos de gênero de internação, são pessoas idosas na faixa etária 60-79 anos, quais os implicadores? renda? raça? local de residencia? em discussão há a necessidade de elucidar o fortalecimento do trabalho pela APS com pessoas idosas. Em resumo incluir número total da amostra, revisar ao longo do texto e substituir idoso (a) por pessoa idosa (o), terminologia mais recente.

